# Balance equations can buffer noisy and sustained environmental perturbations of circadian clocks

**DOI:** 10.1098/rsfs.2010.0007

**Published:** 2010-12-01

**Authors:** Mirela Domijan, David A. Rand

**Affiliations:** Warwick Systems Biology and Mathematics Institute, University of Warwick, Coventry CV4 7AL, UK

**Keywords:** balance equations, environmental perturbations, circadian clocks, mathematical models, sensitivity analysis, robustness

## Abstract

We present a new approach to understanding how regulatory networks such as circadian clocks might evolve robustness to environmental fluctuations. The approach is in terms of new balance equations that we derive. We use it to describe how an entrained clock can buffer the effects of daily fluctuations in light and temperature levels. We also use it to study a different approach to temperature compensation where instead of considering a free-running clock, we study temperature buffering of the phases in a light-entrained clock, which we believe is a more physiological setting.

## 1. Introduction

**Figure 1.**
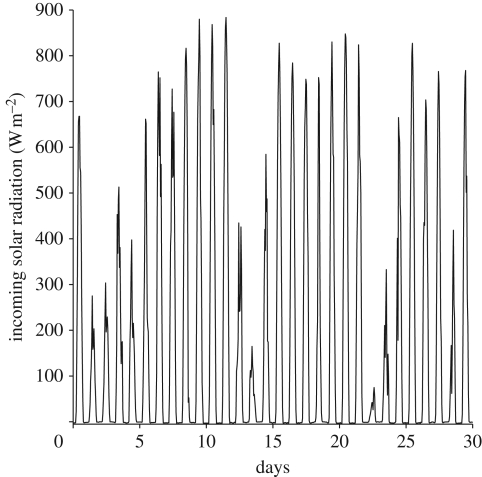
Light intensity measurements for the month of September 2005 from the environmental radiometry data from Harvard Forest [17].

**Figure 2.**
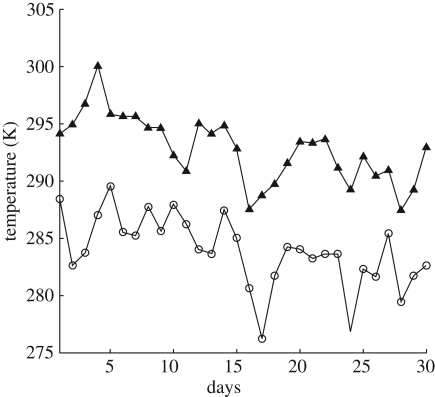
Daily maximum and minimum temperature measurements for the month of September 2005 from UK Met Office Historical Central England data (HadCET) [18].

Circadian oscillators are entrained by the daily cycles of light and temperature. It is therefore important that a clock is sensitive to their daily periodicity. On the other hand, there are very substantial stochastic day-to-day fluctuations in these environmental cycles. This can, for example, be seen in the time series for light intensity and temperature shown in figures 1 and 2. The daily fluctuations in both are substantial: the fluctuations in light have a coefficient of variation of approximately 36 per cent, while those of England's maximum and minimum temperatures in degrees centigrade over just the single month of September have a coefficient of variation 15 and 29 per cent, respectively.

The presence of such substantial noisy perturbations raises a number of questions. Our theoretical understanding suggests that in a typical model these perturbations will produce substantial fluctuations in the protein levels (e.g. figure 4). This seems to be in conflict with the need for the clock to provide robust signals to the genes that it is controlling. It therefore raises the question of whether the clock can be designed so that the daily protein variation is robustly entrained but at the same time the system manages to effectively buffer the stochastic variations.

In this paper, we explain how it is relatively easy for evolution to adjust a clock network so as to carry out such buffering of stochastic environmental fluctuations. We argue that this is potentially an important reason why an oscillator, rather than a direct measurer of the entraining signal (light or temperature) is used. The mathematical argument that we employ is very general and can be applied to a much broader class of regulatory and signalling systems and other environmental factors. It uses a combination of ideas behind balance equations (used previously to explain temperature compensation [1–3]) and the principal component aspects of global sensitivity analysis [4–8]. We consider fluctuations in the light level and temperature fluctuations. To give examples of the effectiveness of the buffering mechanism for light fluctuations, we use a published model of the *Arabidopsis thaliana* circadian clock [7], and for temperature fluctuations, we introduce a temperature-dependent version of this model.

A related issue was addressed in a recent paper [8]. In a study involving artificial *in silico* evolution of clock networks, it was shown that a combination of the need to cope with multiple photoperiods and stochastic variation in the timing of dawn and dusk favoured the evolution of more loops and light inputs and greater complexity in the networks.

After considering short-term fluctuations, we turn to temperature compensation. Temperature compensation refers to the striking and defining feature of circadian clocks whereby their period only varies by a small amount over a physiological range of temperatures [9,10]. However, the exact value of the free-running period in constant conditions does not appear to have a direct selective value in the natural environment, as the clock will normally be entrained to diurnal day/night cycles. One may therefore ask why temperature compensation has arisen during evolution. We address this question here and develop a theory to address the question of how evolution might act on forced entrained oscillators. We show that for typical systems, the phases of the proteins in the clock will vary significantly with temperature but that one can tune the clock to satisfy certain balance equations so that the changes in phase are buffered. We propose that the buffering of the free-running period characteristic of classically temperature-compensated systems is a consequence of this phase buffering. The idea that there is a connection between the free-running period and the entrained phase is not a new one. The way in which phase changes as one crosses an Arnold tongue is well understood in dynamical systems theory [5,11] and in circadian rhythms [12,13]. This relation between the period and the entrainment phase has been observed experimentally in physiologically relevant situations [14].

Throughout, we assume that our circadian clock is modelled by a set of ordinary differential equations1.1
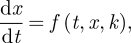
where *t* is time, a vector *x* = (*x*_1_, … , *x*_*n*_) represents the state variables (namely, mRNA and protein levels) and *k* = (*k*_1_, … , *k*_*s*_) is a vector of parameter values. We assume that equation (1.1) has an attracting periodic solution *x* = *g*(*t*,*k*) of period *T*. We study the properties of this solution. We will illustrate our results by using a model of the *Arabidopsis* circadian clock [7].

## 2. Global sensitivity analysis and its principal components

If *g*(*t*) is the solution of equation (1.1) mentioned above, then the change *δg*(*t*) in *g* caused by a change *δk* = (*δk*_1_, … , *δk*_*s*_ ) in the parameter vector *k* is
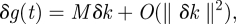
where the linear map *M* is given by
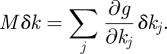
We can regard *M* as a map from the parameter space ℝ^*s*^ to a space of time series. For our purposes, the appropriate space of time series is the Hilbert space *ℋ* defined in appendix A, and we also consider the subspace *ℋ*_0_ of *ℋ* spanned by the functions ∂*g*/∂*k*_*j*_(*t*) on 0 ≤ *t* ≤ *T*, *j* = 1, … , *s*.

In Rand [6], it is shown that there are a set of numbers *σ*_1_ ≥ *σ*_2_ ≥ … ≥ *σ*_*s*_, a set of orthonormal vectors *V*_1_, … ,*V*_*s*_ of the parameter space ℝ^*s*^ and a set of orthonormal vectors *U*_1_, … ,*U*_*s*_ in *ℋ* such that *MV*_*i*_ = *σ*_*i*_*U*_*i*_, M*U_*i*_ = *σ*_*i*_*V*_*i*_ with the following optimality property: for all *k* ≥ 1, the average error given by
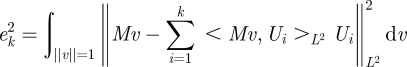
is minimized over all orthonormal bases of *H*_0_. At this minimal value, *e*_*k*_^2^ = *cσ*_*k*_^2^, where *c* is an absolute constant. The *σ*_*i*_ are uniquely determined and the *V*_*i*_ and *U*_*i*_ are, respectively, eigenvectors of MM* and M*M. Thus, the *σ*_*i*_ are the eigenvalues of M*M. If they are simple eigenvectors, then the *U*_*i*_ and *V*_*i*_ are uniquely determined.

M* is the adjoint to *M* and is given by
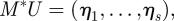
where
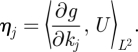
It follows that the *ij*th element of M*M is given by
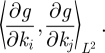
Since this is self-adjoint, it has real positive eigenvalues and these are *σ*_1_ ≥ *σ*_2_ ≥ … ≥ *σ*_*s*_.

**Figure 3.**
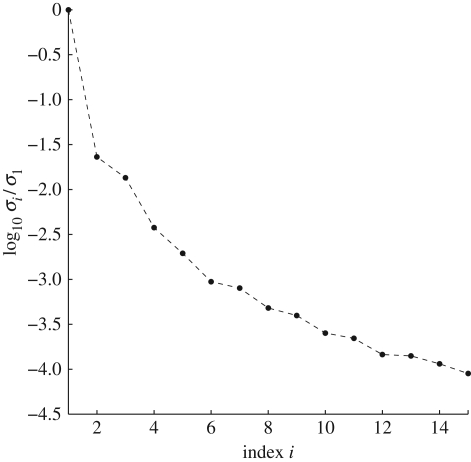
Plot of log_10_(*σ*_*i*_/*σ*_1_) for the largest singular values of the clock model [9]. The *σ*_*i*_ decay exponentially.

It follows from the above discussion that a change *δk* to the parameters leads to a change *δg* in the solution *g* such that
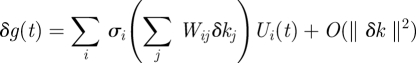
and that the *σ*_*i*_ decay as rapidly as is possible for any such representation. Here, the *W*_*ij*_ are the elements of the inverse matrix *W* = *V*^−1^. The speed with which the *σ*_*i*_ decay for the *Arabidopsis* clock model [7] is shown in figure 3. Inspection of figure 3 shows that this decay is exponential for the case for the clock model of Locke *et al.* [7], and this is typical [4,6,15] as also shown in the electronic supplementary material, figure S1.

Note that when applying this theory, one can either apply it directly to the model parameters *k*_*j*_ or one can apply it to the parameters *η*_*j*_ = log *k*_*j*_. It is often more relevant to use the second approach because, in regulatory and signalling systems, the values of two parameters may differ by an order of magnitude or more. Therefore, it is more appropriate to consider relative changes in the parameters *k*_*j*_ than the absolute changes. The only change to the theory when considering relative changes instead of absolute ones is to replace the linear operator *M* above by *M* · *Δ*, where *Δ* = diag(*k*) is the diagonal matrix whose diagonal is made up of the parameter values. If we denote quantities for the latter relative case using a tilde, we have *Ṽ* = *Δ* · *V*, *W̃*_*ij*_ = *k*_*j*_*W*_*ij*_ and *σ̃*_*i*_ = *σ*_*i*_. This follows from the fact that for small changes *δk* to the model's parameters, *δη*_*j*_ = *δk*_*j*_/*k*_*j*_. The scaled changes *δη*_*j*_ also have the advantage of being non-dimensional.

## 3. Balance equations for sustained and daily fluctuations

Now suppose that a subset {*k*_*j*_1__, … , *k*_*j*_*q*__} of the parameters depend upon a parameter *p*, i.e. *k*_*j*_*m*__ = *k*_*j*_*m*__(*p*). Then,3.1
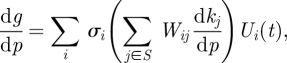
where the latter sum is over *j* in *𝒮* = {*j*_1_, … , *j*_*q*_}. Thus, if we want d*g*/d*p* = 0, we require3.2
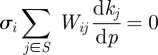
for *i* = 1, … , *s*. The *s* equations in (3.2) are called the *balance equations* and the sums ∑_*j*∈*𝒮*_ *W*_*ij*_ d*k*_*j*_/d*p* are called *balance sums*.

Note that the *i*th balance equation can only be solved if, for this value of *i*, the *W*_*ij*_d*k*_*j*_/d*p* do not all have the same sign. This places a constraint on the *W*_*ij*_ and hence on the system as a whole.

Now we come to the reason why using the above principal components *U*_*i*_ is important. Note that in equation (3.1), each term ∑_*j*∈*S*_ *W*_*ij*_d*k*_*j*_/d*p* is multiplied by *σ*_*i*_ and therefore if the *σ*_*i*_ decrease rapidly, as usually is the case for the sort of systems that we consider, then the importance of the balance equations in ensuring d*g*/d*p* ≈ 0 decreases rapidly as *i* increases, and obtaining the balance equations for just a few low *i* will substantially decrease |d*g*/d*p*|. Inspection of figure 3 shows that this is certainly the case for the clock model of Locke *et al.* [7] and this is typical [4,6,15].

For circadian oscillators, we are often particularly interested in the changes in phase of the various components of the clock. If we define the phase *φ*_*m*_ of the *m*th component as the time that it reaches its maximum value, then [6]3.3
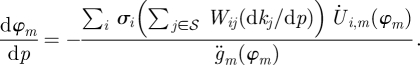
A derivation of equation (3.3) is provided in appendix A. Thus, the balance equation to obtain d*φ*_*m*_ /d*p* ≈ 0 agrees with equation (3.2).

In practice, the balance sums are never zero, and the aim of the balancing is to reduce them substantially in the sense that the ratio of the sum after balancing to that before is substantially less than one.

## 4. Application to daily light fluctuations

Our aim here is to demonstrate a simple mechanism that can enable the clock to filter out the sort of substantial variation in daytime light intensity that is observed in the Harvard Forest data [17], figure 1.

We denote the time-dependent light intensity by *θ*(*t*). For example, *θ*(*t*) might be the function that is 1 between dawn *t*_l_ and dusk *t*_d_ and zero elsewhere or it might be a slightly smoothed version of this discontinuous function. We model fluctuations in the light by replacing *θ*(*t*) by *αθ*(*t*), where *α* fluctuates daily around its mean value of *α* = 1.

We then consider a model where the effect of light is to modulate a subset of the terms in the differential equation so that they are functions of *αθ*(*t*). In general, the light input will occur in multiple terms in the form *k*_*j*_*θ*(*t*), where *k*_*j*_ is one of the parameters of the system. If the light level is fluctuating as above, then the term *k*_*j*_*θ*(*t*) is replaced by *k*_*j*_*αθ*(*t*). Let *𝒮* be the set of parameter indices for the parameters that occur in this way.

Suppose that this original model is not buffered against variation in daytime light intensity. To buffer it, we can assume that there is some simple regulation of the light inputs, so each of the terms *k*_*j*_*αθ*(*t*), *j* ∈ *𝒮*, is replaced by *k*_*j*_(*c*_*j*_*α* + *d*_*j*_)*θ*(*t*). It is also natural to assume that *c*_*j*_ + *d*_*j*_ = 1 since this implies that *k*_*j*_(*c*_*j*_*α* + *d*_*j*_) fluctuates around *k*_*j*_. One can always reduce to this case by scaling the *k*_*j*_ in advance. We call this the light-modified model.

If *c*_*j*_ and *d*_*j*_ are fixed (and not regarded as parameters), then the new value for *W*_*ij*_ in this new system is *c*_*j*_*W*_*ij*_, where the latter *W*_*ij*_ is the value in the original system. Therefore, the balance equation for absolute changes in the parameters is4.1
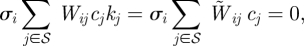
where the *W̃*_*ij*_ are as above for the approach where one uses relative changes in parameters instead of absolute ones so that one uses *η*_*j*_ = log *k*_*j*_.

This is a very general formulation and is, for example, directly applicable to the model of Locke *et al.* [7] for the *Arabidopsis* clock that introduces light in the way described. The light parameters *k*_*j*_, *j* ∈ *𝒮*, are listed in table 1. The light parameters that have the greatest effect on the balance equations (by ranking *σ*_*i*_*W*_*ij*_) are LHY transcription (*q*_1_), light and TOC1-mediated induction (*q*_2_ and *n*_4_) of Y transcription and accumulation of protein P (*p*_5_).

**Table 1.** Left: parameters for the balanced model. Each light parameter *k*_*j*_ has light intensity of the form *α*_*j*_*L*(*t*). For the Locke model, *α*_*j*_ = *α* = 1, while for the balanced model, *α*_*j*_ = *c*_*j*_*α* + *d*_*j*_, where *c*_*j*_ are listed in the table and *d*_*j*_ are *d*_*j*_ = 1−*c*_*j*_. Right: the sum ∑_*j*_*W*_*ij*_d*k*_*j*_/d*α* for *i* = 1,…, 8 evaluated for the Locke model (d*k*_*j*_/d*α* = 1) and the balanced model (d*k*_*j*_/d*α* = *c*_*j*_). The corresponding singular values *σ*_*i*_ are plotted in figure 3.

*k*_*j*_	*c*_*j*_	∑_*jεS*_*W*_*ij*_ (d*k*_*j*_/d*α*)		
		*i*	(d*k*_*j*_/d*α*) = 1	(d*k*_*j*_/d*α*) = *c*_*j*_
*q*_1_	1.1918			
*n*_0_	1.2	1	0.0002	−0
*m*_5_	−314.2308	2	0.0094	0
*m*_7_	1.0163	3	−0.0158	−0
*q*_2_	1.2491	4	0.0466	−0.0117
*n*_4_	−0.2427	5	−0.0213	0.0154
*p*_5_	−0.6042	6	−0.0453	0.0140
*q*_3_	1.5	7	0.0032	0.0033
*q*_4_	1.0198	8	−0.0314	0.0116

**Figure 4.**
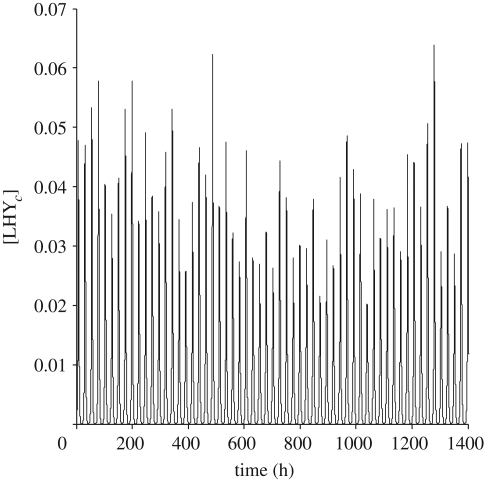
Effect of light intensity fluctuations on the protein profile of the morning loop component LHY of the Locke model. The profile reflects levels of the cytoplasmic protein for light intensity *α* ∼ *𝒩*(1,0.2).

We assume that the variations *α* are normally distributed with mean *μ* and standard deviation *σ*, which we denote by *α* ∼*𝒩*(*μ*,*σ*). As shown in figure 4 (and in the electronic supplementary material, figure S2), the daily variation in the amplitude of the limit cycle solution of this model is substantial and quantitatively reflects the variation in the light amplitude. A light-modified model was constructed as described for which the left-hand sides of equation (4.1) are substantially smaller, as is given in table 1.

**Figure 5.**
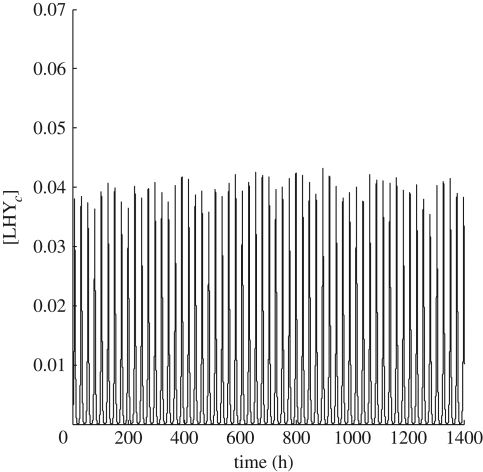
Effect of light intensity fluctuations on the morning loop protein LHY of the balanced model. The profile reflects levels of the cytoplasmic protein for light intensity *α* ∼ *𝒩*(1,0.2).

To implement this, the values of six of the *c*_*j*_ were chosen fairly arbitrarily but relatively close to one and then the remaining three were calculated by solving the linear balance equations corresponding to the first three principal components. Our balanced model shows less variation in the output protein and gene levels (figure 5 for *α* ∼ *𝒩*(1,0.2)). For all genes and proteins, the balanced model shows less variation in the concentration levels than the unbalanced Locke model (electronic supplementary material, figure S3 and table S1). The phases of the balanced model show less variation for all components, except the LHY gene and proteins, which are of same order as those of the Locke model (electronic supplementary material, table S2).

## 5. Application to daily temperature fluctuations

We consider a model with fixed but differing day and night temperatures, *T*_D_ and *T*_N_. Below, we derive a set of balance equations that eliminate the effects of temperature fluctuations.

We assume that the temperature enters the model through a set of parameters *k*_*j*_, *j* ∈ *𝒮*, which are temperature sensitive. A standard assumption for the temperature dependence of each of model parameters *k*_*j*_ is that it is similar to that for rate constants of chemical reactions and is described by the Arrhenius equation. This expresses the dependence of the rate constant *k*_*j*_ on the temperature *T* and activation energy *E*_*j*_ as *k*_*j*_ = *A*_*j*_ exp(−*E*_*j*_/*RT*), where *A*_*j*_ is a constant specific to the individual parameter and *R* is the gas constant (8.314472 × 10^−3^ kJ mol^−1^K^−1^). In our case, we need to deal with the fact that we have different night and day temperatures and therefore we assume5.1
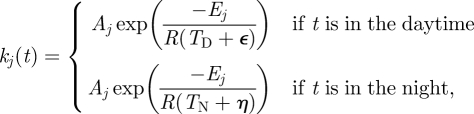
where *t* is in the daytime if *t*_l_ ≤ *t* mod *τ* < *t*_d_, where [*t*_l_, *t*_d_] denotes the range of day hours, and otherwise *t* is in the night. Moreover, *ε* and *η* denote the fluctuations in day and night temperatures, *T*_D_ and *T*_N_, respectively. We consider the following first-order Taylor series expansion:5.2
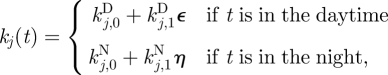
where *k*_*j*,0_^D^ = *A*_*j*_ exp(−*E*_*j*_/*RT*_D_) and *k*_*j*,1_^D^ = − *k*_*j*,0_^D^*E*_*j*_/*RT*_D_ and similarly for the night parameters. Note that this is a very good approximation and higher order terms can be neglected, since for a parameter of order *𝒪*(0.1) (i.e. the order of a large number of Locke parameters), with sensible activation energy *E* ≈ 50 kJ mol^−1^, the coefficient of the second-order term is of order *𝒪*(0.001).

From the observation in equation (3.1), day temperature variations *ε* will have the following effects on changes to the solution *g*:5.3
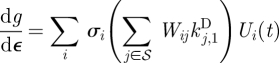
and the following changes to the phases:5.4
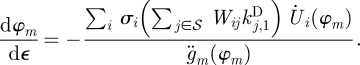


Similar expressions can be derived for night temperature fluctuations. Together, these give rise to two sets of balance equations,5.5
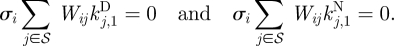
Or alternatively,5.6
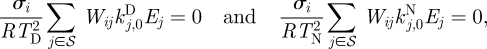
since, by the above, *k*_*j*,1_^D^ = −*k*_*j*,0_^D^*E*_*j*_/*RT*_D_^2^. In order to make a model temperature dependent, we have to define the dependence of its parameters on temperature. It is likely that all parameters in a regulatory system are actually temperature dependent, but this temperature dependence will have little effect for a parameter *k*_*j*_ if all the sensitivities *S*_*ij*_ = *σ*_*i*_*W*_*ij*_ are small for that value of *j*. Therefore, in order to keep the model reasonably tractable, we will only introduce temperature into those variables with a significant sensitivity.

**Figure 6.**
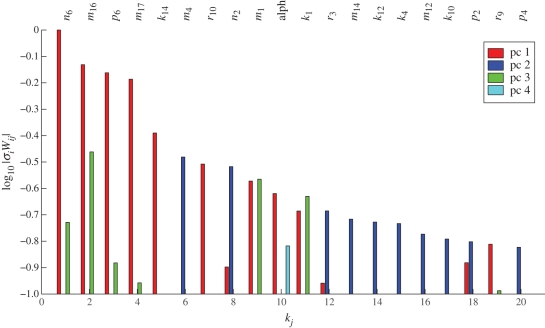
Power sensitivity spectrum of the Locke model. Each group of bars corresponds to the values of log_10_|*σ*_*i*_*W*_*ij*_| for a parameter *k*_*j*_. These are only plotted for those *i* for which log_10_|*σ*_*i*_*W*_*ij*_| is significant (here that is *i* = 1–4). The parameters *k*_*j*_ are ordered by max_*i*=1−4_ log_10_|*σ*_*i*_*W*_*ij*_| and only 20 most sensitive ones are plotted. Not taking into account Hill terms *g*_2_ and *g*_7_ (for reasons outlined in the main text), parameter *p*_6_ has the highest max_*i*=1−4_ log_10_|*σ*_*i*_*W*_*ij*_|. Only seven other parameters (parameters up to and including *m*_1_) have sensitivity higher than 30 per cent of the maximum, and they will be candidates for temperature-sensitive parameters.

Moreover, to determine the relative importance of parameters, since values of some parameters differ by an order of magnitude or more, it is more appropriate to compare relative changes of parameters and to use the log parameters and the *W̃*_*ij*_ as described in the last paragraph of §2. We selected parameters that ranked highest when ordered by max_*i*=1−4_ |*σ*_*i*_*W̃*_*ij*_|. The plot of top 20 is shown in figure 6. In this selection, we include only parameters that come linearly in the model and exclude Hill coefficients as these are not rate constants. Parameter *n*_6_ has the highest max_i=1−4_log_10_|*σ*_*i*_*W̃*_*ij*_|. Only seven other parameters have sensitivity higher than 30 per cent of the maximum. These eight parameters will be temperature sensitive.

Note that the key parameters to control the buffering were parameters linked to PRR7/9 components, namely LHY-dependent transcription (*n*_6_), mRNA degradation (*m*_16_), translation (*p*_6_) and cytoplasmic protein degradation (*m*_17_) as well as the nuclear-cytoplasmic transport (*r*_12_). Aside from these, the list also includes TOC1 light-independent transcription (*n*_2_), mRNA degradation (*m*_4_) and LHY mRNA degradation (*m*_1_). Each of the parameters *k*_*j*_ was separated into a morning and an evening term, *k*_*j*_ = *k*_*j*_^D^*θ* + *k*_*j*_^N^(1 − *θ*), where morning and evening terms were adjusted to be a fixed percentage higher or lower than the Locke model values. Note that the term *θ* is the light function. For most parameters, we chose this to be 10 per cent, but for *m*_17_, we had to choose a significantly smaller value of 1 per cent, so that gene and protein waveforms of the temperature-sensitive model would match those of the Locke model as closely as possible. We confirmed that the parameters had a strong effect on the model by verifying that several of them appeared in the top 10 per cent of parameters ordered by their magnitude of max_*i*=1−4_log_10_|*σ*_*i*_ *W̃*_*ij*_|. We chose night and day temperatures to be *T* = 285.15 K and *T* = 290.15 K, to be close to the temperature data from UK Met Office (figure 2), with mean maximum and minimum temperatures *T* = 292.79 K and *T* = 283.92 K.

We calculated the energies of the new model and checked the balance equations. We tried several different combinations of energies, then recalculated the morning and evening parameters and then computed the balance equations for the model. From the starting Locke model, we evolved two models with differing values of energies (table 2). We labelled them a balanced and an unbalanced model according to their fit to the balance equations (table 3).

**Table 2.** Energies and night and day parameter values for the unbalanced and the balanced model. Note that day temperature *T*_D_=290.15 K and night temperature is *T*_N_=285.15 K.

	unbalanced			balanced		
*k*_*j*_	*E*_*j*_	*k*_0,*j*_^D^ (*T*_D_)	*k*_0,*j*_^N^ (*T*_N_)	*E*_*j*_	*k*_0,*j*_^D^ (*T*_D_)	*k*^N^_0,*j*_(*T*_N_)
*n*_6_	13.7721	7.6670	8.4742	27.6101	7.2635	8.8777
*m*_16_	39.1133	11.0158	14.6380	27.6091	11.0158	13.4638
*p*_6_	41.5837	0.2471	0.3343	27.6374	0.2616	0.3198
*m*_17_	2.7490	4.4595	4.5495	2.7490	4.4595	4.5495
*m*_4_	13.7658	3.6320	4.0142	27.6079	3.4408	4.2054
*r*_10_	27.5836	0.1991	0.2433	27.5836	0.1991	0.2433
*n*_2_	27.6115	2.7078	3.3096	27.6115	2.7078	3.3096
*m*_1_	27.6087	1.7991	2.1989	27.6087	1.7991	2.1989

**Table 3.** The balance sums ∑_*j*_*W*_*ij*_*k*_0,*j*_^D^ *E*_*j*_ and ∑_*j*_*W*_*ij*_*k*_0,*j*_^N^ *E*_*j*_ and the corresponding singular values *σ*_*i*_ for the unbalanced model (UB) and the balanced model (B). To get the true sums, divide each column by 1/*RT*^2^, where *T*_D_=290.15 K and *T*_N_=285.15 K.

	∑_*j*_*W*_*ij*_*k*_*i*,1_^D^(*T*_D_)		∑_*j*_*W*_*ij*_*k*_*i*,1_^N^(*T*_N_)		*σ*_*i*_ (× 10^4^)	
*i*	UB	B	UB	B	UB	B
1	−0.1031	0.0380	−0.0215	−0.0082	1.0349	1.5089
2	−0.7195	0.4218	−0.2216	−0.0488	0.0402	0.0361
3	−6.3831	8.8031	−1.0568	1.0984	0.0182	0.0114
4	0.9973	−2.7543	−3.6876	0.0877	0.0069	0.0051
5	2.2247	2.2007	−0.0972	2.6921	0.0024	0.0031

The balanced model was chosen so that originally it had activation energies of about 30 kJ mol^−1^ for almost every component except for that of the parameter *m*_17_ whose activation energy was chosen to be significantly lower to make balancing easier. Since later we adjusted the temperature range to fit with the data seen in figure 2, these values appeared slightly smaller. The unbalanced model was made by changing four energies from the list of the balanced model, in order to make a worse fit to the balance equations.

**Figure 7.**
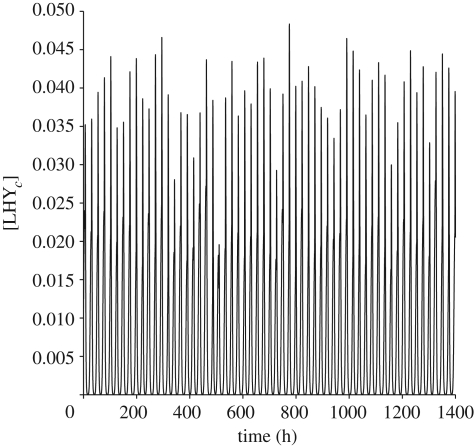
Effect of night temperature fluctuations on protein profile of the morning loop component LHY of the Locke model. The profile reflects levels of the cytoplasmic protein for night temperature variations *η*, *ε* ∼ *𝒩*(0,1).

**Figure 8.**
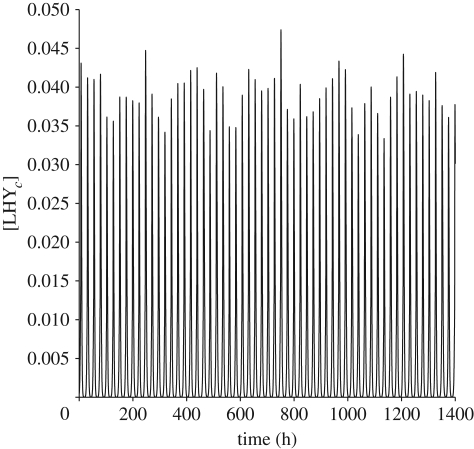
Effect of night temperature fluctuations on the morning loop protein LHY of the balanced model. The profile reflects levels of the cytoplasmic protein for night temperature variations *η*, *ε* ∼ *𝒩*(0,1).

In fact, our initial temperature-sensitive model gave the best balance equations, so it was chosen as the balanced model. The balanced model shows less variation in peak concentrations and phase variations than the unbalanced model (figures 7 and 8 and electronic supplementary material, tables S3 and S4).

## 6. Application to temperature compensation

Instead of temperature compensation on a free-running clock (i.e. d*p*/d*T* ≈ 0), we are interested in temperature compensation of the light-entrained clock, which we believe is a more physiological setting. The aim is to minimize protein and phase changes in the context of changing temperature, d*φ*/d*T* ≈ 0. We therefore consider models that are entrained to the day–night cycle rather than the free-running clock.

This hypothesis [16] that a balance of opposing reactions could allow temperature compensation was first tested by Ruoff using a simple model for an oscillatory feedback loop [1–3]. He used an Arrhenius representation for temperature dependence and deduced a balance equation for the local period slope d*p*/d*T* in terms of the activation energies *E*_*j*_ and control coefficients for each of the parameters.

We also assume that parameters *k*_*j*_, *j* ∈ *𝒮*, are temperature dependent and describe them by Arrhenius equations, *k*_*j*_ = *A*_*j*_ exp(−*E*_*j*_/*RT*), as described above. The temperature *t* is in kelvins in the range 285.15 K ≤ *T* ≤ 300.15 K. Activation energies, *E*_*j*_, must be in the range 1 kJ mol^−1^ ≤ *E*_*j*_ ≤ 150 kJ mol^−1^. We insist that some of the activation energies are substantial because otherwise the parameters only have weak dependence upon temperature. In fact, for a given balanced system, since these energies enter linearly into the balance equation, scaling them by a factor just scales the divergence from perfect balance by that factor.

We now apply equation (3.2) where *p* is replaced by temperature *T*. From the relation d*k*_*j*_ /d*T* = *k*_*j*_*E*_*j*_ /*RT*^2^, we deduce that the balance equations are6.1
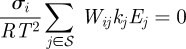
for *i* = 1, …, *s*. When the *σ*_*i*_ decrease rapidly, we need only consider these balance equations for the first few *i* in order to get d*g*/d*T* or d*φ*_*m*_/d*T* small. Note that the *i*th equation can only be solved if, for this value of *i*, the *W*_*ij*_ do not all have the same sign. This is because *k*_*j*_ *E*_*j*_ is always positive. This places a constraint on the *W*_*ij*_ and hence on the system as a whole. Only certain networks can be balanced.

We assume that the model parameters from Locke *et al.* [7] correspond to a model at *T* = 288.15 K. Since this model does not include temperature, we selected the temperature-sensitive parameters at each temperature end by checking the parameter sensitivity spectrum, as described in §5.

We consider three models in which temperature dependence is inserted into the Locke model. In two of them (models 1 and 2), the parameter values at *T* = 288.15 K are as in the original model but although they have the same activation coefficients *A*_*j*_, they have different activation energies *E*_*j*_ (table 4).

**Table 4.** Energy values *E*_*j*_ of the temperature-sensitive models based on the Locke *et al.* [7] model: model 1 (M1) and model 2 (M2).

*k*_*j*_	*E*_*j*_ (M1)	*E*_*j*_ (M2)
*n*_6_	11.3399	22
*m*_16_	12.3650	5
*p*_6_	7.5775	7.5775
*m*_17_	10.2539	10.2539
*r*_10_	48.3543	48.3543
*m*_4_	42.2986	10
*n*_2_	50.8636	50.8636
*m*_1_	1.2201	1.2201

The energies for model 1 were selected so that they were substantial and roughly satisfied the balance equation (table 6). We then iteratively calculated the *W*_*ij*_'s for the new model and more exactly rebalanced the equation. The iterative procedure converged reasonably well. For model 2, the energies were modified so that the balance equations were not well satisfied (table 6). Some energies were decreased as well as increased (table 4). We could easily do this as the two models share *W*_*ij*_'s at *T* = 288.15 K.

**Figure 9.**
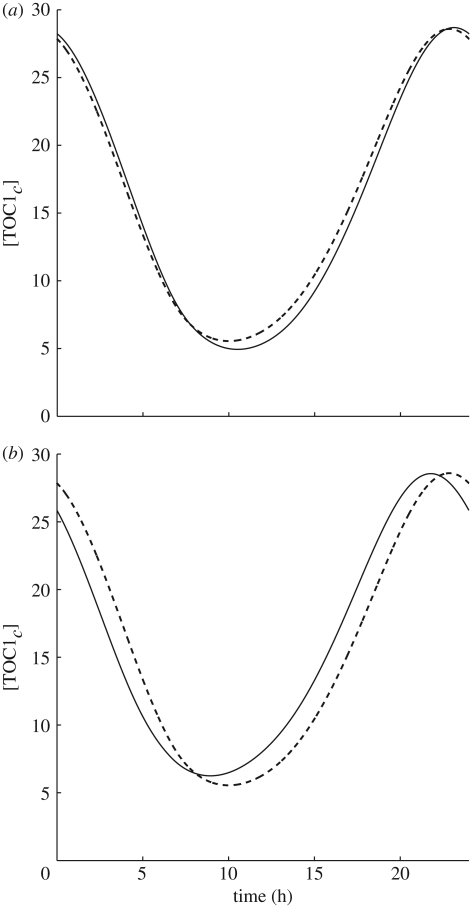
TOC1 protein profiles. The Locke model at *T* = 288.15 K (dashed line) against the balanced model 1 at *T* = 289.15 K (*a*) and the unbalanced model 2 at *T* = 289.15 K (*b*). By balancing, we can ensure minimum change to protein profile and phases.

**Figure 10.**
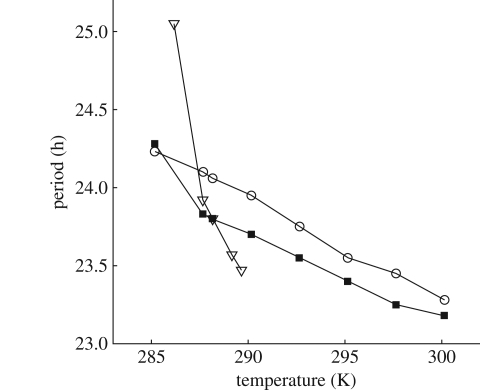
Period of the free-running temperature-sensitive models. The plot shows the period of model 3 (open circles) and the Locke temperature-sensitive model with different energies, models 1 and 2 (black squares and open triangles, respectively). The original model of Locke *et al*. [7] is plotted as the third black square (*T* = 288.15 K). Both temperature-sensitive Locke models cannot temperature compensate as well as model 3. Model 2 is arrhythmic outside the range plotted.

The temperature compensation in model 2 is substantially worse than that in model 1, demonstrating the importance of balancing. We find that model 1 is better at local temperature compensation than model 2, with less variation in its protein concentrations and phases from the original published model [7], cf. TOC1 protein times series in figure 9, and also it fares better at global temperature compensation (electronic supplementary material, tables S8 and S9). Moreover, model 1 can temperature compensate in constant light conditions (figure 10), with model 2 becoming arrhythmic outside a narrow temperature range. Model 3 shares activation energies of model 1 but has different activation coefficients *A*_*j*_. Thus, its parameter values do not agree exactly with the Locke model at any temperature (table 5). This model is better balanced than either of the other two (table 6) and is significantly better compensated for phase than the other models (tables 7 and 8 and electronic supplementary material, table S10). Moreover, in continuous light conditions, its period is much better compensated than model 2 and slightly better than model 1 (figure 10).

**Table 5.** Values of temperature-dependent parameters for model 3 with *T*_0_ = 285.15 K and *T*_1_ = 300.15 K.

*k*_*j*_	*E*_*j*_	*k*_*j*_ (*T*_0_)	*k*_*j*_ (*T*_1_)
*n*_6_	11.3399	7.6784	9.7517
*m*_16_	12.3650	11.5927	15.0445
*p*_6_	7.5775	0.2812	0.3299
*m*_17_	10.2539	4.2545	5.2810
*r*_10_	48.3543	0.2050	0.5000
*m*_4_	42.2986	3.0918	8.5677
*n*_2_	50.8636	2.4062	7.0300
*m*_1_	1.2201	1.9883	2.0401

**Table 6.** Sums ∑_*jε*𝒮_*W*_*ij*_*k*_*j*_*E*_*j*_ at each temperature *T*_0_ = 285.15 K, *T*_1_ = 288.15 K and *T*_2_ = 300.15 K for all three models. To get the true sums, divide each sum by 1/*RT*^2^. The corresponding singular values *σ* are shown in parentheses.

		∑_*jε*𝒮_*W*_*ij*_*k*_*j*_(*T*)*E*_*j*_ and (*σ*_*i*_ (× 10^4^))		
	*i*	*T*_0_	*T*_1_	*T*_2_
model 1	1	0.0397	0.0419	−0.0229
		(18.8826)	(1.0648)	(1.2884)
	2	−1.1872	0.0134	0.1168
		(0.0261)	(0.0352)	(0.0367)
model 2	1	0.0840	−0.0787	0.0544
		(4.8274)	(1.0648)	(3.6128)
	2	1.0042	−0.2398	0.1579
		(0.0325)	(0.0352)	(0.0310)
model 3	1	0.0434	0.0286	0.0162
		(1.2388)	(1.5245)	(1.2037)
	2	−0.0681	0.1220	0.1057
		(0.0368)	(0.0351)	(0.0368)

**Table 7.** Peak and trough times of cytoplasmic protein at temperatures *T*_0_ = 285.15 K and *T*_1_ = 300.15 K of model 3 (the globally compensated model) with parameter values from table 5.

	peak times		trough times	
	(*T* = *T*_0_)	(*T* = *T*_1_)	(*T* = *T*_0_)	(*T* = *T*_1_)
LHY_*c*_	7.2	7.11	20.7	19.7
TOC1_*c*_	22.8	22.2	9.6	10.0
X_*c*_	5.0	4.5	15.7	15.7
Y_*c*_	6.9, 16.0	6.9, 16.8	1.8, 10.0	2.2, 9.8
PRR7/9_*c*_	16.9	16.9	23.2	23.0

**Table 8.** Peak and trough times of cytoplasmic protein of model 2 at temperatures *T*_0_ = 285.15 K and *T*_1_ = 300.15 K.

	peak times		trough times	
	(*T* = *T*_0_)	(*T* = *T*_1_)	(*T* = *T*_0_)	(*T* = *T*_1_)
LHY_*c*_	7.1	8.2	19.8	21.9
TOC1_*c*_	23.1	13.1	9.5	23.5
X_*c*_	5.3	20.1	15.9	5.0
Y_*c*_	6.9, 16.4	2.2, 7.0	2.4, 9.8	6.0, 17.5
PRR7/9_*c*_	16.0	16.9	0	2.5

## 7. Discussion

We have attempted to show how a combination of ideas behind balance equations and the principal component aspects of global sensitivity analysis gives a new approach to understanding how to design regulatory networks that are buffered against certain fluctuating environmental perturbations. As well as presenting some concrete examples of applications to circadian clocks, we have described the general theory behind this. From this, it is clear that it can be applied to a much broader class of regulatory and signalling systems and environmental factors other than light and temperature.

If buffering of a particular environmental fluctuation has significant selective pressure for the organism in question, then we can interpret this selective pressure as acting on the balance equation. There will then be selective pressure on the quantities that make up the left-hand side of the equation (e.g. system parameters, activation energies) to make them balance to zero. Understanding this makes it much clearer how evolution can act to achieve what appear to be quite complex tasks.

Temperature compensation has been one of the driving dogmas of circadian biology and has been interpreted in terms of the constancy or near-constancy of the free-running period of the circadian clock under changing temperature. However, it is not clear how evolution acts on the free-running period since in physiological conditions the clock is entrained to the day–night cycle and has a period of 24 h. We show that through certain balance equations it is possible to buffer the changes in phase over relatively large ranges of temperature. Although we do this for a specific example, the mathematical approach suggests that this buffering should be possible for an extremely broad range of clock models. We suggest that the observed near-constancy of the free-running period is a consequence of the near-constancy of the phases of the entrained clock.

To balance a balance equation, it is necessary that the terms making up the equation do not all have the same sign. For example, for temperature compensation, we require that the relevant quantities *W*_*ij*_ do not all have the same sign. This puts constraints on the network structure, and this leads to a prediction about what network structures can be expected.

## 8. Methods

All the computations were carried out using Matlab and XPPAUT. In particular, the global sensitivity calculations were done using the Matlab-based Time Series Sensitivity Analysis Package available from http://www2.warwick.ac.uk/fac/sci/systemsbiology/software/, and the period calculations were performed using XPPAUT, available from http://www.math.pitt.edu/~bard/xpp/xpp.html.

## Appendix A

### A.1. Definition of Hilbert space *ℋ*

This is the *L*^2^ Hilbert space of ℝ^*n*^-valued functions *U*(*t*) = (*U*_1_(*t*), … ,*U*_*n*_(*t*)), *U*^′^(*t*) = (*U*^′^_1_(*t*), … ,*U*^′^_n_(*t*)), 0 ≤ *t* ≤ *T*, with inner product

and norm given by ||*U*||^2^_*L*^2^_ = 〈*U*,*U* 〉_*L*^2^_.

### A.2. Derivation of equation (3.3)

This was presented in Rand [6]. Let *t* = *ϕ*_*m*_(*k*) be the time when the concentration of the *m*th component, *g*_*m*_, is at a maximum or a minimum value. Hence, *ϕ*_*m*_(*k*) satisfies *ġ*_*m*_(*ϕ*_*m*_(*k*),*k*) = 0. Differentiating both sides with respect to *k*_*j*_ and rewriting,
